# Age and Race Disparities in Viral Suppression and the Moderating Effect of Substance Use Among Men Who Have Sex with Men Living with HIV

**DOI:** 10.1007/s11414-025-09948-0

**Published:** 2025-05-09

**Authors:** Ran Fang, Jake C. Steggerda, Deborah Konkle-Parker, Andrew C. Voluse

**Affiliations:** 1 Department of Psychiatry and Human Behavior, University of Mississippi Medical Center, Jackson, MS USA; 2 Department of Psychiatry, University of Florida, Gainesville, FL USA; 3 Schools of Nursing, Medicine and Population Health, University of Mississippi Medical Center, Jackson, MS USA

## Abstract

Viral suppression is essential for individuals living with HIV, as it is linked to improved clinical outcomes and long-term health. Research has documented age and racial disparities in HIV viral suppression. Men who have sex with men (MSM) are particularly affected by HIV infections, especially in the Southern United States. Studies indicate that substance use among people with HIV in the U.S. presents significant barriers to engaging in HIV care. This study investigated the relationships between age, race, MSM status, and viral suppression among men living with HIV (MLWH), who participated in the Helping HAND program at an academic medical center in a Southern state. The analysis included 746 male participants, primarily Black/African American. The results showed that increases in age were positively associated with a greater likelihood of viral suppression, even after adjusting for harmful or hazardous drinking, problematic substance use, race, and MSM status. Younger MSM participants were less likely to achieve viral suppression than older MSM participants. In this male only sample, neither race nor MSM status was found to be related to viral suppression. Additionally, harmful or hazardous drinking and problematic substance use did not moderate the associations between age, race, or MSM and viral suppression. These findings highlight disparities in viral suppression across different age groups among men living with HIV. The results emphasize the need for targeted outreach initiatives specifically designed for younger age cohorts living with HIV, including MSM.

## Introduction

In the era of HIV treatment as prevention, viral suppression is essential in patients living with HIV [1]. Research has demonstrated that HIV viral suppression predicts improved clinical outcomes such as morbidity and mortality [2]. Previous studies suggest viral suppression is closely related to long-term outcomes, including progression to AIDS and life expectancy [3]. In addition, viral suppression reduces HIV transmission through sexual contact [4–6].

Age and racial disparities in HIV viral suppression have been well documented. It is estimated that nearly 50% of people living with HIV (PLWH) in the US are older than 50 years of age [7]. However, research shows that only 67% of individuals achieved viral suppression in the oldest age group (aged 55 years and older) of people living with HIV in the US, whereas younger people had lower viral suppression, ranging from 60.3 to 66.2% in ascending order among age groups 13–24, 25–34, 35–44, and 45–54 [8]. In the Ryan White HIV/AIDS Program (RWHAP), which supports health care and support services for more than 50% of all PLWH in the United States, researchers have demonstrated that younger clients had lower viral suppression, whereas clients aged above 65 years showed the highest viral suppression [9]. Previous research indicates that Black people were disproportionately influenced by lower viral suppression than other racial groups [10, 11].

A wealth of literature supports racial/ethnic disparities in viral suppression. A recent study in the Los Angeles County Medical Care Coordination Program suggests persistent racial/ethnic disparities after accounting for nine psychosocial domains, such as housing, substance use, and mental health levels [12]. Of 8979 participants consisting of diverse racial/ethnic groups, the authors disclose lower baseline percentage of viral suppression in Black/African Americans (49.5%), compared to White patients (53.5%) [12]. The RWHAP, administered by the Health Resources and Services Administration (HRSA), which served more than 551,500 clients in 2016, reveals that the disparity between Black or African American patients and White patients was 8.1%, although the gap had reduced from the year 2010 [9]. In a cross-sectional study among 12,394 HIV patients in the United States, a diverse sample consisting of Black, Hispanic, and White indicates that White men had higher prevalence of viral suppression than other subgroups [13]. Given the importance of viral suppression, more study is needed to identify correlates, particularly among the vulnerable populations, that contribute to the development of effective prevention and intervention methods.

Although men who have sex with men (MSM) represent approximately 3.9% of the U.S. population [14], they are disproportionately affected by HIV infections, particularly in the south [15, 16]. Black MSM living with HIV are more likely to be virally non-suppressed than White MSM. An early study using the Medical Monitoring Project (MMP), a national HIV surveillance system, reports lower viral suppression among Black MSM than White MSM living with HIV [17]. A community-based cohort study of Black and White MSM living with HIV in Atlanta Georgia concludes that Black MSM were significantly associated with lack of viral suppression, compared to White MSM [18]. Relatedly, findings from the MMP report higher percentage of a suppressed viral load among MSM than men who have sex with women, based on various sexual risk behaviors [19]. Furthermore, data from the National HIV Surveillance System (NHSS) among the 38 jurisdictions has found that viral suppression was highest among MSM, compared to other transmission categories [20]. These inconsistent findings highlight the importance of geographic differences in interpretating the relationship between Black MSM living with HIV and viral suppression. As such, further study is needed to look at modifiable factors that can influence viral load in the south among Black MSM.

Substance use disorders (SUDs) are common among people with HIV in the United States and serve as severe barriers to engagement in HIV care [21–23]. Men and Black individuals of younger age are disproportionately affected by SUDs [23]. The research results vary in relation to the correlation between substance use disorders and the ability to suppress viral load among individuals with HIV. In a study examining the association between substance use and viral suppression by age and race, researchers demonstrate that substance use was negatively associated with viral suppression among MSM aged 25–34 years and for Black MSM [21]. It has been reported that among PLWH, substance use, regardless of behavior patterns (i.e., persistent or intermittent use), was a significant predictor of non-viral suppression, compared with persistent non-users [24]. A recent study shows that recent substance use or history of SUD was independently and positively associated with less likelihood of viral suppression [25]. However, a longitudinal HIV cohort study in the District of Columbia during 2011–2018 reveals that SUDs disproportionately affected Black individuals living with HIV but were not directly associated with engagement in care, HIV medication prescription, or viral suppression [23]. Importantly, no linear relationship has been found between harmful or hazardous drinking and viral suppression. Recent literature using the 3-item Alcohol Use Disorders Identification Test Consumption (AUDIT-C) with scores ranging from 0 to 12 reveals that only medium alcohol drinking (women = 3–5, men = 4–5) was predictive of a decreased likelihood of viral suppression [26]. In a large sample study, it has demonstrated that the majority of fluctuations in alcohol consumption were not significantly linked to viral suppression [27]. Therefore, the current literature does not offer comprehensive understanding of the connections between alcohol drinking and viral suppression among PLWH. Further study is needed to clarify the role of substance use in the associations of age, race, and MSM status with viral suppression.

### The current study

A significant gap has been a lack of extant research exploring associations between age, race, and viral suppression among PLWH or MSM in the American South. The purpose of the current study was to examine associations between age, race, MSM status, and viral suppression, and whether these associations were moderated by substance use in the male only population living with HIV. Based on prior research, the authors hypothesize that (1) increases in age will increase the likelihood of viral suppression, (2) Black participants will be less likely to achieve viral suppression than White participants, (3) likelihood of viral suppression will significantly differ between MSM and non-MSM groups (i.e., non-MSM will have smaller likelihood of viral suppression than MSM), and (4) harmful or hazardous drinking and problematic substance use will moderate the associations between demographic characteristics (i.e., age and race) and viral suppression. Given the importance of viral suppression and its role in morbidity and mortality among PLWH and MSM, knowledge of the present study’s aims is fundamental to developing effective screening and intervention strategies (e.g., target outreach) for these vulnerable populations.

## Methods

### Participants and procedures

Participants were recruited in a federal Ryan White Care Act-funded project called “Helping to Advance in New Directions” (Helping HAND) at an academic medical center in a Southern state [28]. The project aimed at lowering implementation barriers to medical and mental health treatment for PLWH. A large urban HIV clinic serving approximately 2000 PLWH per year (63% male and 85% African American), which was part of the academic medical center, provided brief mental health interventions for those who screened positive for a higher risk of alcohol- and/or drug-related difficulties.

The present study collected data between February 2018 and February 2020 on a web-based software platform, Research Electronic Data Capture (REDCap), hosted by the academic medical center. During this period, 1942 individuals participated in a brief, self-administered tablet computer-based screen that aligns with the conventional screening, brief intervention, and referral treatment (SBIRT) framework [29]. A summary of the screening results was generated and shared with the patient by a trained provider who conducted a brief negotiated intervention and offered linkage to subsequent in-house evidence-based treatment for alcohol and/or drug use for those who yielded positive screening results. Inclusion criteria for the current study included that participants were ≥ 18 years old, self-identified as male (either cisgender or transgender), and provided complete data for the study variables, including age, race, and MSM status. Exclusion criteria included being < 18 years old, not self-identifying as male, and providing incomplete data.

The Institutional Review Board of the University of Mississippi Medical Center approved the data release for the current study.

### Measures

#### Viral load

Viral load was collected upon participants’ routine visit to the HIV clinic using standard laboratory procedures. The authors defined viral suppression as the achievement of a viral load of fewer than 200 copies of HIV RNA/mL, as consistent with the existing literature [30–32]. The authors analyzed their initial lab results upon entering into the study.

#### The alcohol use disorders identification test (AUDIT)

The AUDIT is a 10-item screening tool assessing alcohol consumption, drinking behaviors, and alcohol-related problems during the past year [33]. The questionnaire is a psychometrically validated measure with a Cronbach’s alpha of.93 [34] and has been utilized extensively across different cultures [35]. The self-report measure is scored on a 0–4 scale, with a higher total score indicating more problematic drinking. A score of 8 or more is associated with harmful or hazardous drinking.

#### The drug abuse screening test (DAST)

The DAST is a 10-item brief screening tool assessing for problematic drug use [36]. DAST can be utilized in clinical settings and for assessing treatment outcomes. The estimated internal consistency was reported to be 0.86 to 0.94. [37]. Each question requires a yes or no response to a statement related to potential involvement with drugs, not including alcohol or tobacco use, in the past 12 months. Total scores range from 0 to 10, with higher scores suggesting higher degrees of drug-related problems. A score of 3 or more is associated with problematic substance use.

#### Data analytic plan

Analyses were conducted using SPSS version 27 (IBM Corp, 2020) and R version 4.2.2 (R Core Team, 2022). Of 1942 participants, 1214 identified as male, and 468 did not meet inclusion criteria and were excluded, resulting in 746 participants in the final sample. Analyses were conducted on subsets of the dataset containing male participants only who identified as MSM (*n* = 349) and male participants who did not identify as MSM (*n* = 397). Male participants with missing MSM data (*n* = 468) were excluded from analyses. The authors did not identify a pattern for participants who did not have MSM data, compared to participants who had MSM data. The authors addressed the first hypothesis of the current study by calculating the association between viral suppression (i.e., 0 = non-viral suppression; 1 = viral suppression) and age, adjusting for harmful or hazardous drinking, problematic substance use, race (i.e., White vs. Black), and MSM status using logistic regression analyses. The authors also tested whether there were significant differences in viral suppression across age groups (i.e., 18–24, 25–34, 35–49, 50–60, and 61+), using separate ANOVA analyses. The same analytical approach was used to test the second and third hypotheses, with the only difference being that the associations of viral suppression with race and MSM status were being examined. To address the fourth hypothesis, the authors tested whether the associations between demographic characteristics (i.e., age and race) and viral suppression were moderated by continuous variables of harmful or hazardous drinking and problematic substance use from the AUDIT and/or DAST, using logistic regression analyses.

## Results

### Sample characteristics

Table 1 shows the subsample of 746 individuals included in the current study who identified as male. The sample consisted of predominantly Black/African American participants (*n* = 639, 85.7%), compared to White individuals (*n* = 107, 14.3%). No other races or ethnicities were reported other than Black or White. The mean age was 48.0 years (SD = 11.1), and the largest two age groups were 35–49 (*n* = 306, 41.0%) and 50–60 (*n* = 199, 26.7%), accounting for approximately 68% of the sample. In contrast, only 6 individuals were in the age group of 18–24 (0.8%). The mean scores on the AUDIT-10 and DAST-10 were 3.6 (SD = 5.4) and 1.2 (SD = 1.8), respectively, suggesting both are in the low-risk range.

**Table 1:** Characteristics of participants

Variable		Mean (SD)	n (%)
Age		48.0 (11.1)	
	*18–24*		6 (0.8%)
	*25–34*		126 (16.9%)
	*35–49*		306 (41.0%)
	*50–60*		199 (26.7%)
	*61* +		69 (9.2%)
	*Missing*		40 (5.4%)
Race			
	*White*		107 (14.3%)
	*Black*		639 (85.7%)
MSM Status			
	*MSM*		349 (46.8%)
	*Not MSM*		397 (53.2%)
AUDIT-10		3.6 (5.4)	
DAST-10		1.2 (1.8)	

*MSM*: Men who have sex with men; *AUDIT-10*: Alcohol Use Disorder Identification Test (range = 40, median = 1); *DAST-10*: Drug Abuse Screening Test (range = 10, median = 1)

### Study analyses

The associations of three predictors (age, race, and MSM) with viral suppression are presented in Table 2. Increases in age in the male only sample were positively associated with an increased likelihood of viral suppression when adjusting for harmful or hazardous drinking, problematic substance use, race (White vs. Black), and MSM status (Hypothesis 1). Race was not significantly associated with viral suppression (OR = 0.60, 95% CI [0.17, 1.64]; Hypothesis 2). Similarly, MSM status was unrelated to viral suppression (OR = 1.46, 95% CI [0.33, 5.92]; Hypothesis 3). For non-MSM participants, there were no significant differences in viral suppression at different age categories, *F* (4, 362) = 2.05, *p* =.087. For MSM participants, results indicated that viral suppression significantly differed by age group, *F* (4, 334) = 2.78, *p* =.027. Post hoc pairwise comparisons revealed that participants in the 25–34 age group were less likely to achieve viral suppression than participants in the 35–49 age group (*M*diff = −0.12, *p* =.022), 50–60 age group (*M*diff = −0.13, *p* =.027), and 61+ age group (*M*diff = −0.29, *p* =.012). There were no significant differences among other age groups. Neither harmful or hazardous drinking nor problematic substance use was found to moderate the associations between age, race, or MSM and viral suppression (Hypothesis 4).

**Table 2:** Age, race, and MSM status as predictors of viral suppression: testing whether harmful or hazardous drinking and problematic substance use moderate the associations

	Viral Suppression (N = 746)				
*Predictors*	*OR*	*SE*	*CI*	*z*	*p*
Age	1.43	0.15	1.16–1.77	3.31	.001
Race	0.51	0.19	0.23–1.00	−1.83	.068
MSM Status	1.23	0.26	0.82–1.87	0.99	.321
Harmful/Hazardous Drinking	0.83	0.31	0.41–1.84	−0.49	.625
Problematic Substance Use	0.83	0.21	0.50–1.39	−0.74	.457
Age* Harmful/Hazardous Drinking	1.08	0.13	0.86–1.37	0.68	.498
Age* Problematic Substance Use	1.08	0.13	0.85–1.37	0.59	.554
Race* Harmful/Hazardous Drinking	1.13	0.41	0.52–2.26	0.33	.743
Race* Problematic Substance Use	0.95	0.21	0.61–1.48	−0.21	.836
MSM Status* Harmful/Hazardous Drinking	1.07	0.24	0.69–1.68	0.31	.753
MSM Status* Problematic Substance Use	0.75	0.16	0.49–1.14	−1.34	.181
R^2^ Tjur	0.068				

*OR* = Odds ratio (odds ratios above 1 indicate increased likelihood of viral suppression, odds ratios below 1 indicate decreased likelihood of viral suppression); *SE* = standard error; *CI* = 95% confidence intervals for odds ratios; p-values <.05 are bolded; Race: 0 = White, 1 = Black; *MSM Status*: 0 = non-MSM, 1 = MSM

## Discussion

This is the first known study to examine the moderating role of substance use on viral suppression among a predominantly racial minority male population living with HIV in a Southern state. Overall, increases in age were positively associated with viral suppression among the participant sample. Race was not a significant predictor of viral suppression, likely due to a predominantly Black sample (85.7%). Although MSM status was not a significant of predictor of viral suppression, younger MSM adults aged 25–34 were less likely to achieve viral suppression than older MSM adults.

Early studies have observed a similar trend on the relationship of age and viral suppression—younger PLWH were less likely to achieve viral suppression compared to older PLWH [38, 39]. One explanation of older age being positively associated with higher odds of viral suppression is that antiretroviral therapy (ART) adherence increases with age [40]. Research has demonstrated that an older age was significantly associated with increased functional support (e.g., someone you can confide in), which would link to better ART adherence compared to the absence of support [41]. Furthermore, early research has shown that the older group was significantly less burdened by food restrictions, taking medications, and more optimistic about treatment [42]. Regardless of race, MSM status, and problematic substance use (including alcohol), age in the male only sample was shown to have a significant positive correlation with viral suppression, which is consistent with the extant literature.

The results of the current study reveal disparities in viral suppression in men living with HIV as they age. The current study’s finding that younger MSM had lower likelihood of viral suppression is consistent with other studies examining the association of age with viral load among MSM [21, 43–45]. A large sample study of MSM in the Miami-Dade County Ryan White Program reported that age was an independent predictor of viral suppression [44], which corroborated early findings [38, 39]. Due to small numbers of people aged below 25 years in the current study (<1%), this finding did not apply to the youngest age group. Young MSM bear the full brunt of HIV. Prior epidemiology by individuals study revealed that people aged 25–34 years had the highest diagnosed HIV and the largest percentage increase of diagnoses during the period 2009–2018 from 27 to 36% [46]. In a Southern state, younger MSM in this age group may be more susceptible to additional stressors, including entering the workforce, lack of support, and limited access to healthcare due to financial constraints. The current study adds to extant literature that young MSM, especially young Black MSM, are vulnerable to not achieving viral suppression, even in a clinical setting where these young Black MSM are in care.

The current study did not find a meaningful association between harmful or hazardous drinking and viral suppression, which is consistent with other studies showing no significant associations between the two variables [47–49]. However, an early systematic review indicated that a majority (82%) of the 17 studies observed a negative correlation between alcohol intake and viral suppression, the final and likely most critical component of the HIV continuum of care [50]. In their recent study on changing patterns of alcohol use and viral suppression, the authors found that only increases in drinking from abstinence were associated with a higher risk of viral non-suppression among PLWH [27]. This suggests that the relationship between harmful or hazardous drinking and viral suppression is intricate and warrants further investigation. The overall low mean AUDIT score in the current study sample arguably explains why no association was found.

Additionally, the authors did not find a meaningful relationship between problematic substance use (excluding alcohol) and viral suppression, likely due to relatively low levels of problems related to substance use in the male only study sample, as evidenced by the mean DAST-10 score of 1.2. It is worth noting that studies suggest that individuals with a decreased odd of viral suppression were significantly associated with poly-substance use, being Black, and a younger age [51, 52]. With regard to mechanisms underlying the associations, systematic reviews demonstrate that substance use was among the most highly significant factors influencing ART adherence [53]. As ART adherence plays a crucial role in achieving viral suppression, literature links medication nonadherence to a variety of substances, including stimulants, marijuana, sedatives, and alcohol use [21, 54, 55]

Taken together, the current study’s results indicate that there were no significant moderating effects of harmful or hazardous drinking or problematic substance use (excluding alcohol) on the relationship between age and viral suppression within the predominantly Black cohort in a male population in a Southern state. This suggests strong and consistent associations between age and viral suppression in this demographic.

The findings from the current study should be interpreted with some limitations in mind. First, the current study’s data was comprised of predominantly Black/African American participants (85.7%). In addition, the sample was comprised solely of males in a Southern state. As such, results may not generalize to other population groups. The Helping HAND project serves many PLWH who are uninsured and underinsured. Consequently, findings are not representative of all PLWH or the MSM population. Second, the authors excluded 468 male participants (approximately 38.5% of the total male participants) with no MSM data from the current study, likely decreasing the power of the analysis but unlikely to significantly alter the distribution of responses collected. Third, the authors defined viral suppression as only one viral load test due to the cross-sectional study design, which is not representative of sustained viral suppression. Additional limitations include the biases that exist with self-reported data (e.g., social desirability and recall biases), which may have affected the findings. Future studies recruiting patients with multiple viral load tests are required to validate the findings.

The study nevertheless has several strengths. The authors did not dichotomize harmful or hazardous drinking and problematic substance use, as doing so would reduce the statistical power to detect associations [56]. Additionally, the authors had a relatively large and evenly distributed sample of MSM (*n* = 349) and non-MSM (*n* = 397).

## Implications for Behavioral Health

The findings of this study underscore the need for targeted outreach initiatives specifically designed for younger male cohorts living with HIV, including a younger MSM population. Future studies are needed to investigate the modifiable factors that link age with viral suppression. This includes examining psychological constructs, such as mental health status and coping strategies, as well as health behaviors like medication adherence and access to healthcare services. Understanding how these factors interplay can inform more effective strategies for promoting viral suppression among younger individuals living with HIV. Additionally, the relationship between substance use and viral suppression requires further clarification. Substance use can have significant implications for HIV care, given its disproportionate impact on younger men and Black individuals within this demographic [21–23]. Clarifying this association will be vital for developing comprehensive care approaches that address the unique challenges these populations face. Once a robust understanding of the correlates of viral suppression is achieved, subsequent research should prioritize the development of actionable interventions that can be seamless integrated into clinical practice, enhancing the effectiveness of HIV care delivery. Furthermore, it is critical that policy and funding efforts are strategically directed toward the Southern United States to support above-mentioned work. By focusing resources on these areas, meaningful steps can be taken toward better health for younger men living with HIV.

## Data Availability

The data used in the current study are not available in order to protect the confidentiality of participants.
